# Corrigendum to: Reevaluation of the effect of dietary restriction on different recombinant inbred lines of male and female mice

**DOI:** 10.1111/acel.13534

**Published:** 2021-12-29

Archana Unnikrishnan, Stephanie Matyi, Karla Garret, Michelle Ranjo‐Bishop, David B. Allison, Keisuke Ejima, Xiwei Chen, Stephanie Dickinson and Arlan Richardson, *Aging Cell*, 20, e13500. https://doi.org/10.1111/acel.13500

In the published version of Unnikrishnan et al. (2021), the authors noticed that one of the graph in panel (b) of Figure 1 was wrong. Panel (b) for the male violin plots that was published was the same as the female mice. The correct figure, showing the violin plots for the male 115‐RI mice is shown below, and this does not change the results.

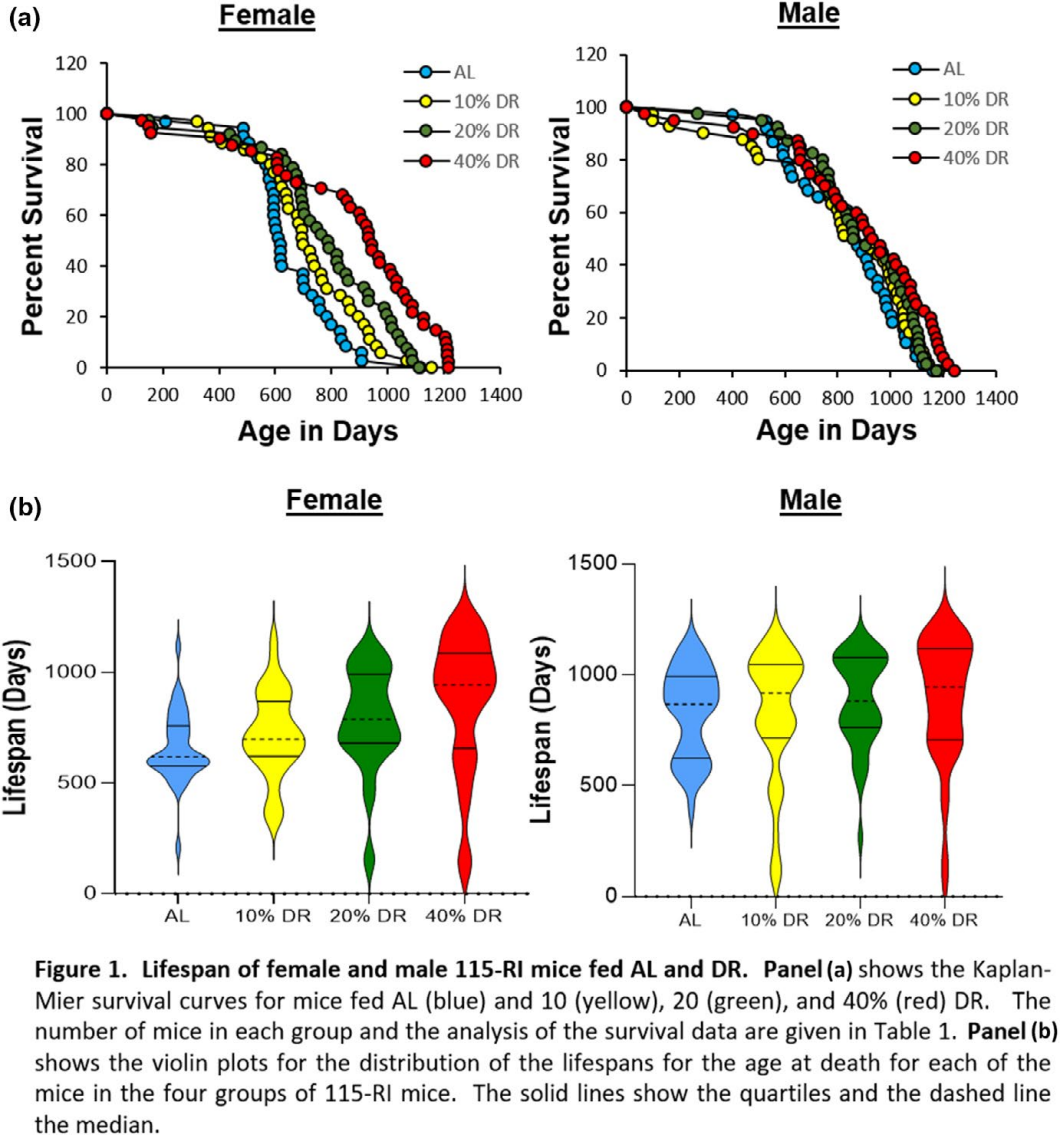

The authors apologize for this error.
