# Supplementary figures and images for: Reevaluation of the effect of dietary restriction on different recombinant inbred lines of male and female mice
Source: Aging Cell. 2021 Oct 29;20(11):e13500. doi: 10.1111/acel.13500 (PMC8590105; doi:10.1111/acel.13500)

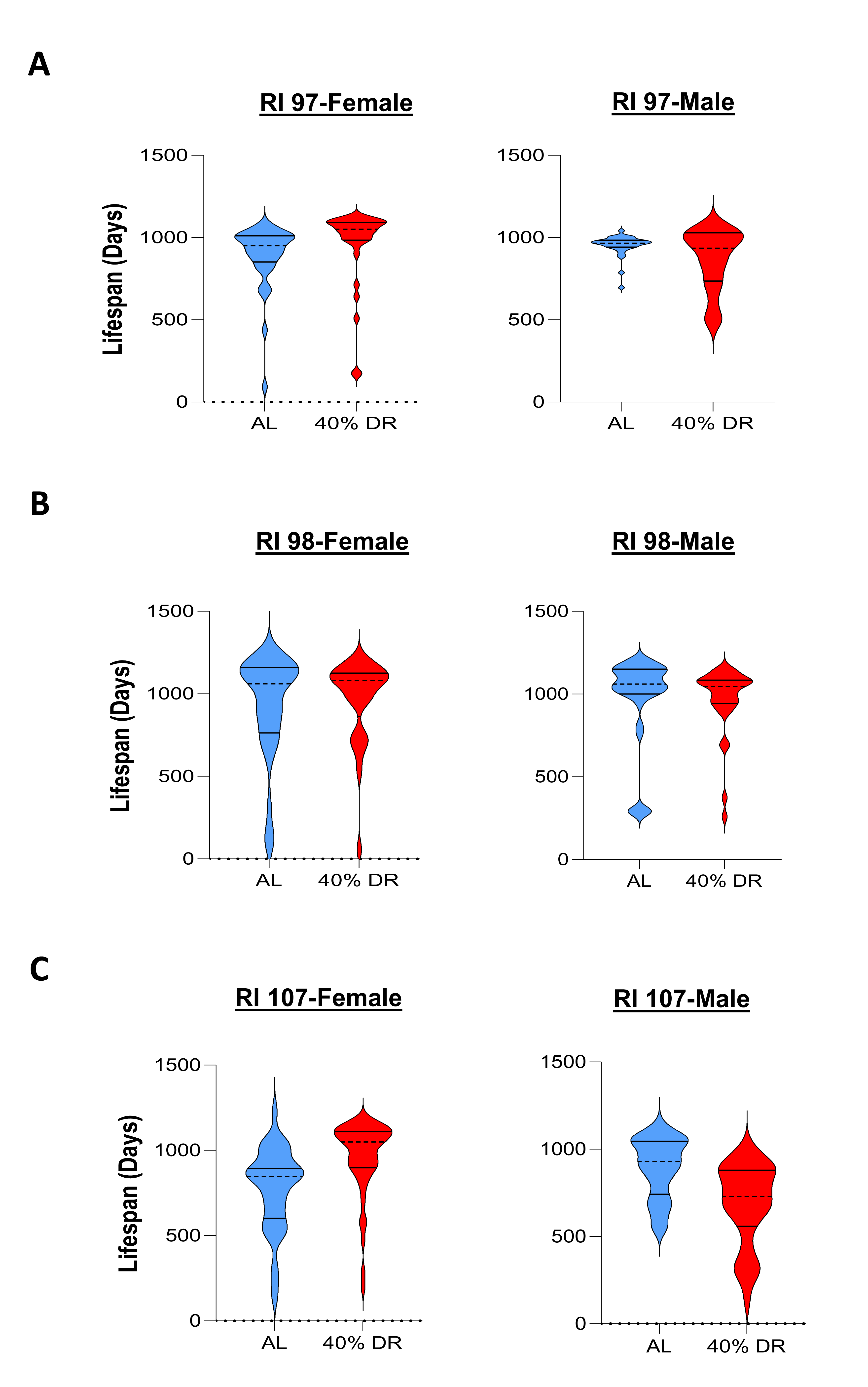

Supplement: Supplementary file 1 — Fig S1 [file ACEL-20-e13500-s001.tif]

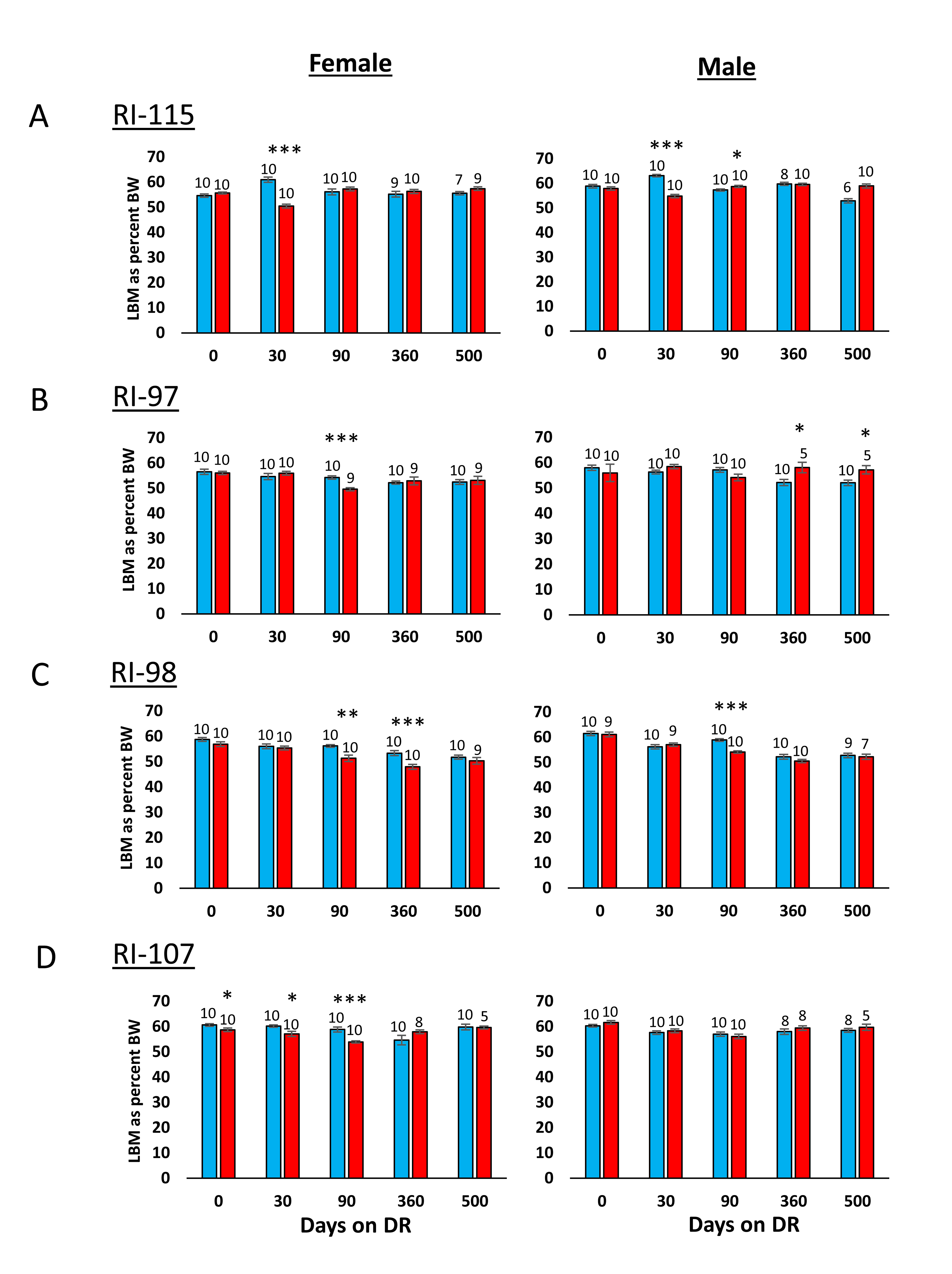

Supplement: Supplementary file 2 — Fig S2 [file ACEL-20-e13500-s003.tif]

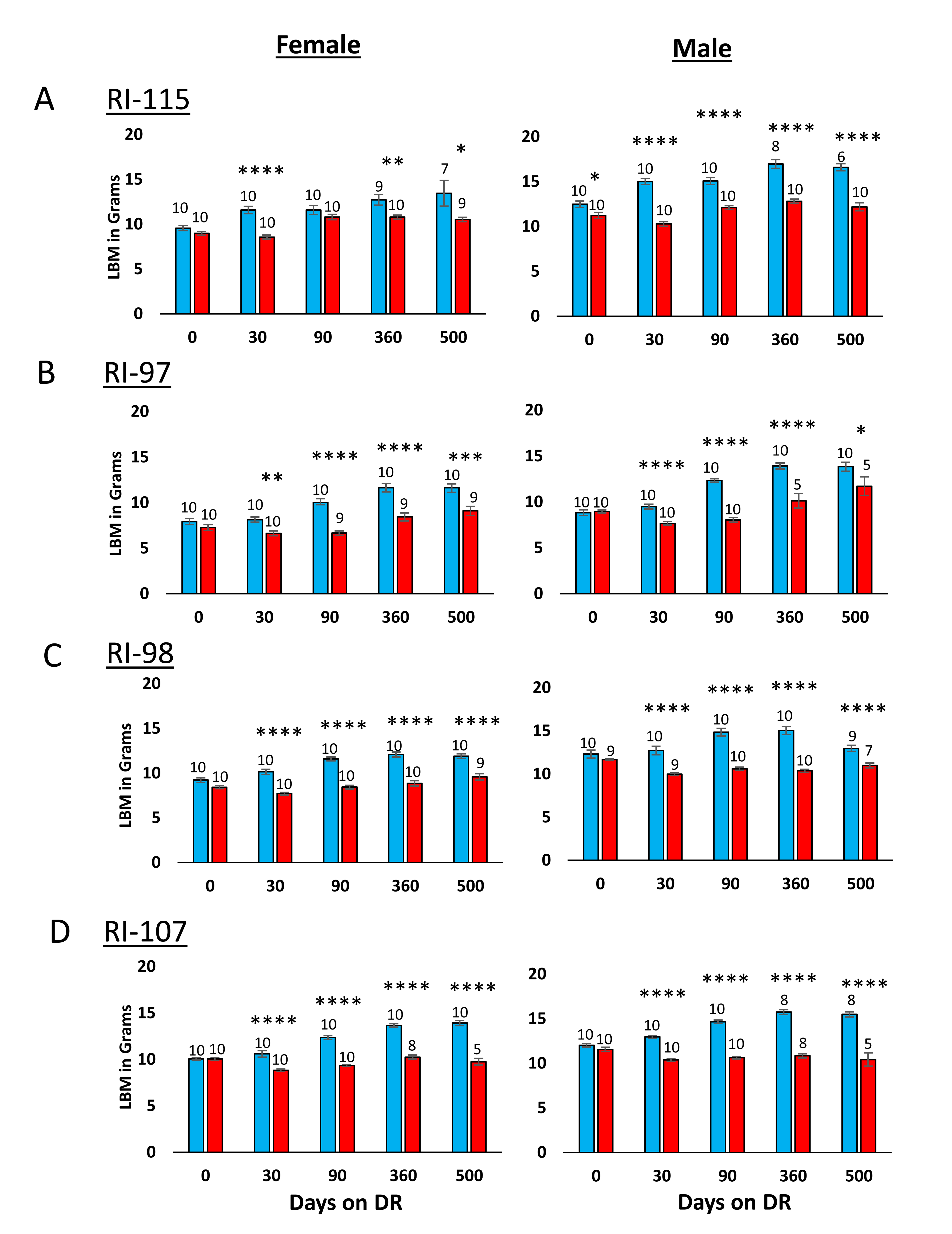

Supplement: Supplementary file 3 — Fig S3 [file ACEL-20-e13500-s006.tif]

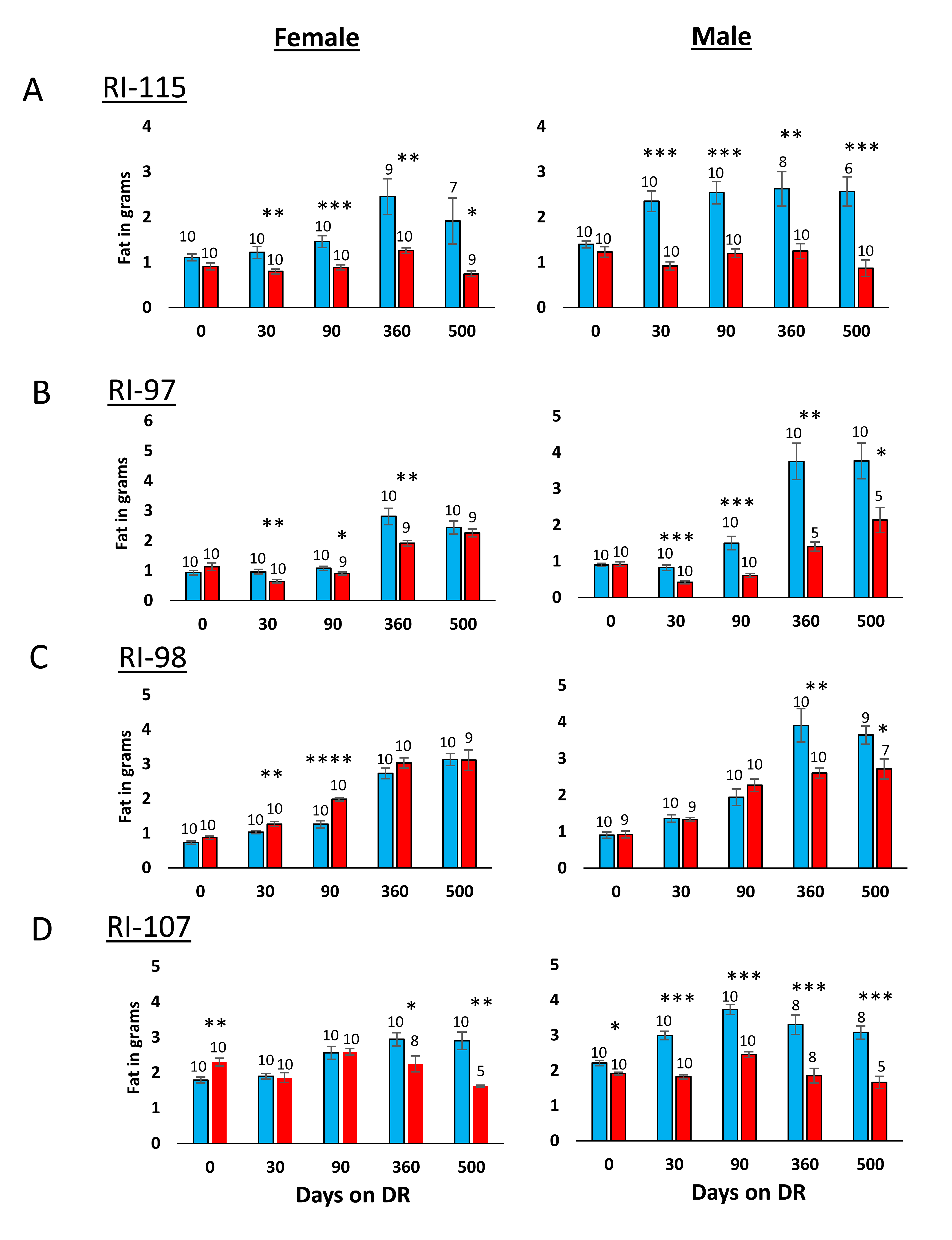

Supplement: Supplementary file 4 — Fig S4 [file ACEL-20-e13500-s005.tif]

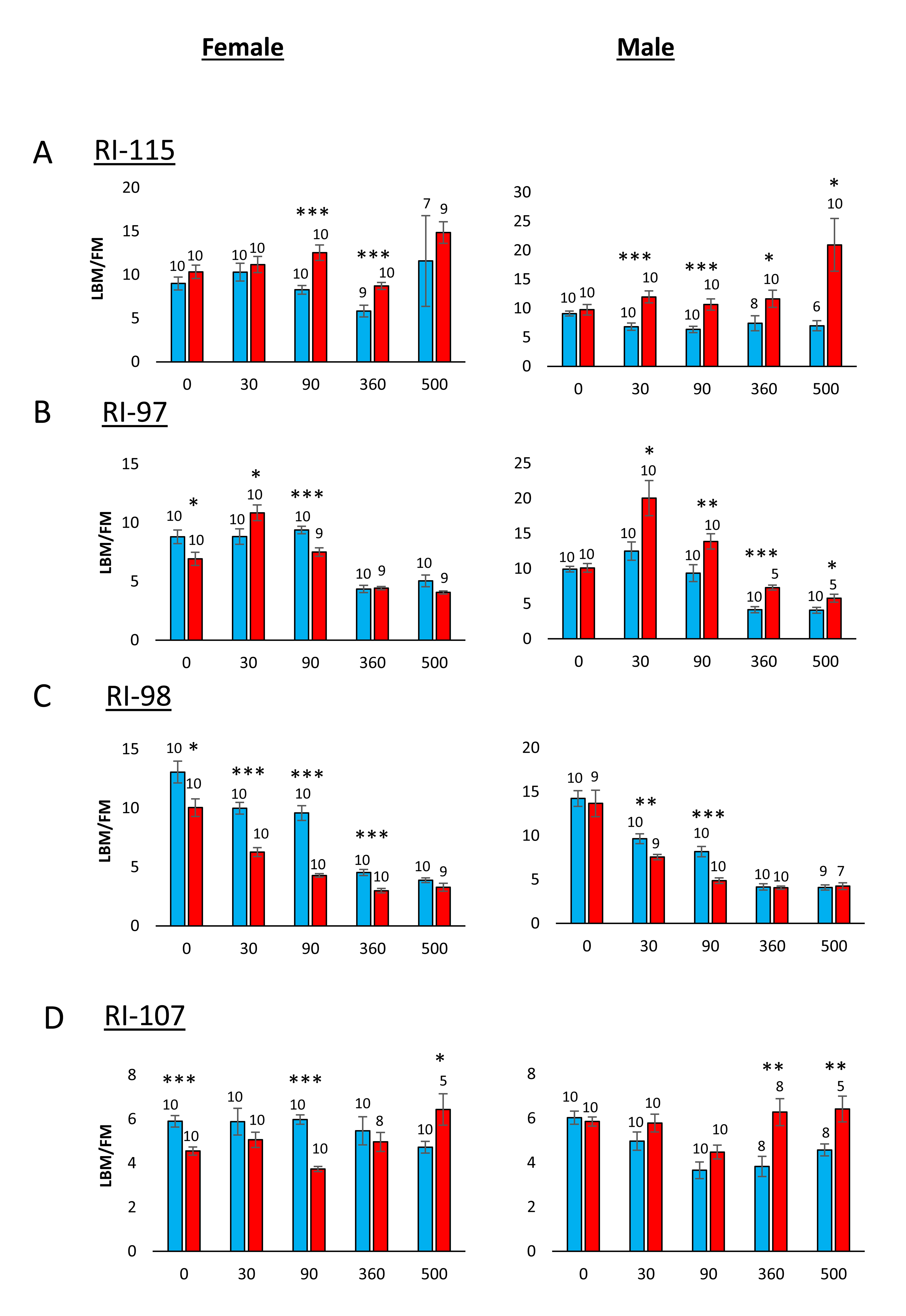

Supplement: Supplementary file 5 — Fig S5 [file ACEL-20-e13500-s007.tif]

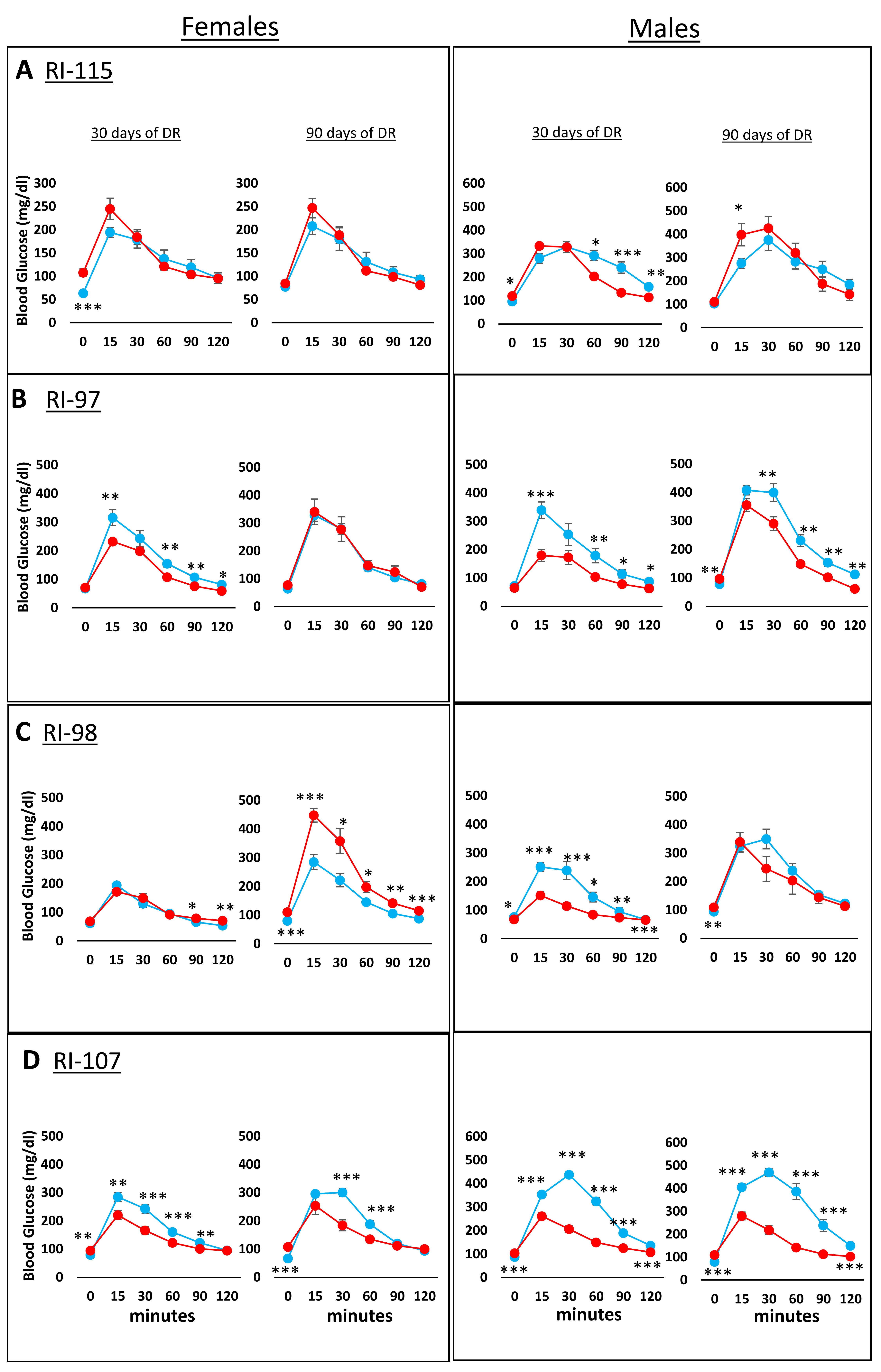

Supplement: Supplementary file 6 — Fig S6 [file ACEL-20-e13500-s002.tif]
