# Supplementary material for: Reevaluation of the effect of dietary restriction on different recombinant inbred lines of male and female mice
Source: Aging Cell. 2021 Oct 29;20(11):e13500. doi: 10.1111/acel.13500 (PMC8590105; doi:10.1111/acel.13500)
Supplement: Supplementary file 7 — Table S1 [file ACEL-20-e13500-s004.docx]

| **Supplemental Table 1. Food Consumption at 6-months of age** | |
| --- | --- |
| **Strain of Mice** | **Food Consumed/mouse/day**  **(grams ± SEM)** |
| **Females** | |
| RI 115 | 3.2 ± 0.1^a^ |
| RI 97 | 2.6 ± 0.1^b^ |
| RI 98 | 2.6 ± 0.1^b^ |
| RI 107 | 2.6 ± 0.1^b^ |
| **Males** | |
| RI 115 | 3.4 ± 0.2^a^ |
| RI 97 | 2.8 ± 0.1^b^ |
| RI 98 | 3.1 ± 0.1^c^ |
| RI 107 | 2.7 ± 0.1^b^ |

Each value represents the mean ± SEM for 30 mice fed over 8 days. For each sex, values that are significant difference between RI-lines (p<0.05 using one-way ANOVA with Tukey’s test) are shown by different letter superscripts.
